# Supplementary material for: A high-temperature double perovskite molecule-based antiferroelectric with excellent anti-breakdown capacity for energy storage
Source: Nat Commun. 2023 Apr 27;14:2420. doi: 10.1038/s41467-023-38007-5 (PMC10140061; doi:10.1038/s41467-023-38007-5)
Supplement: Supplementary file 1 — Supplementary Information [file 41467_2023_38007_MOESM1_ESM.pdf]

Supplementary Information

A High-Temperature Double Perovskite Molecule-Based Antiferroelectric with Excellent Anti-Breakdown Capacity for Energy Storage

Yi Liu, Yu Ma, Xi Zeng, Haojie Xu, Wuqian Guo, Beibei Wang, Lina Hua, Liwei Tang, Junhua Luo, and Zhihua Sun**

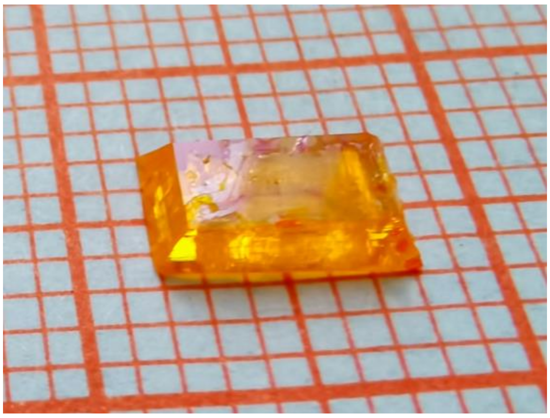

Supplementary Fig. 1 Plate-like single crystal of **1** obtained by the temperature-cooling method.

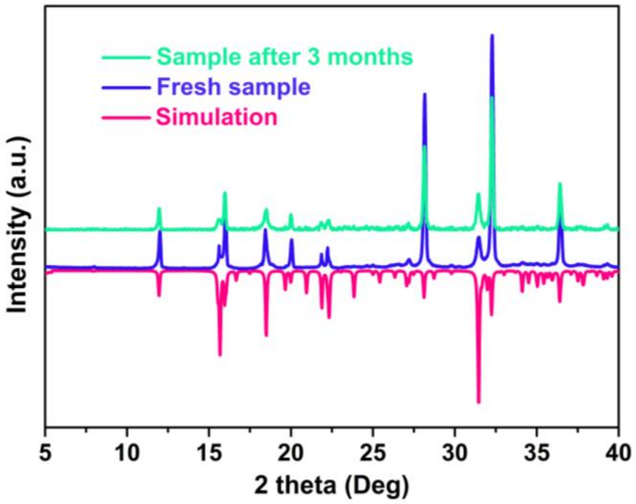

Supplementary Fig. 2 Experimental (fresh sample and sample after 3 months) and simulated PXRD patterns for **1** at room temperature.

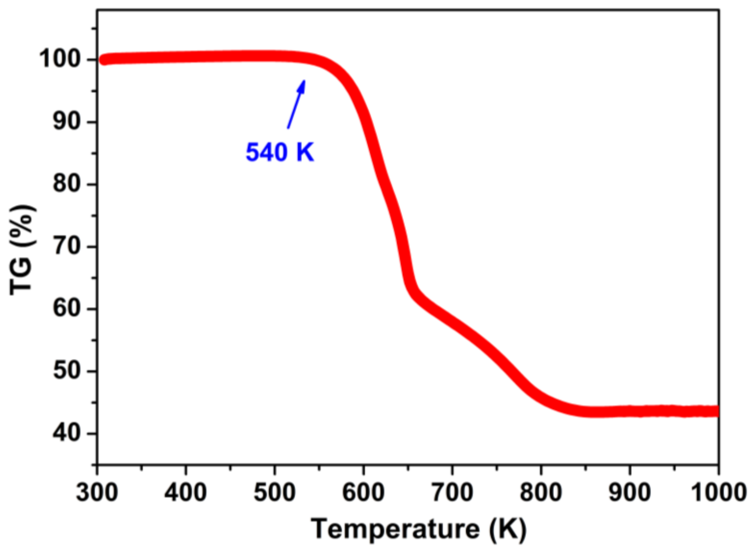

Supplementary Fig. 3 TG result shows that **1** can maintain thermal stability up to ~540 K.

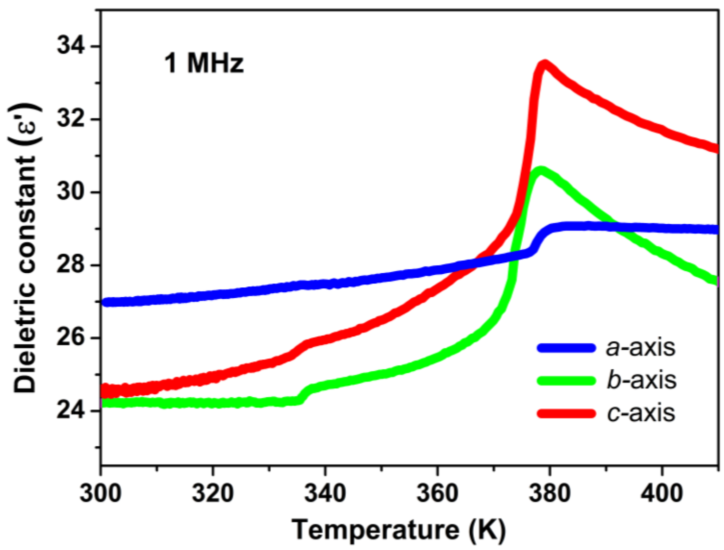

Supplementary Fig. 4 Temperature-dependent ε' measured along with different axis directions at 1 MHz.

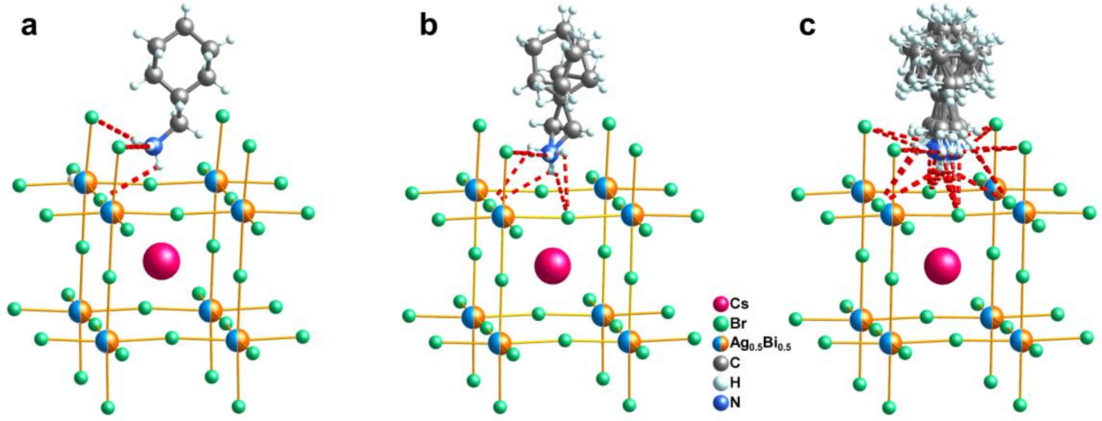

Supplementary Fig. 5 The N-H···Br hydrogen-bonding interactions between organic cations and inorganic perovskite frameworks of **1** at **a** LTP, **b** ITP and **c** HTP. Red dotted lines represent the N-H···Br hydrogen bonds.

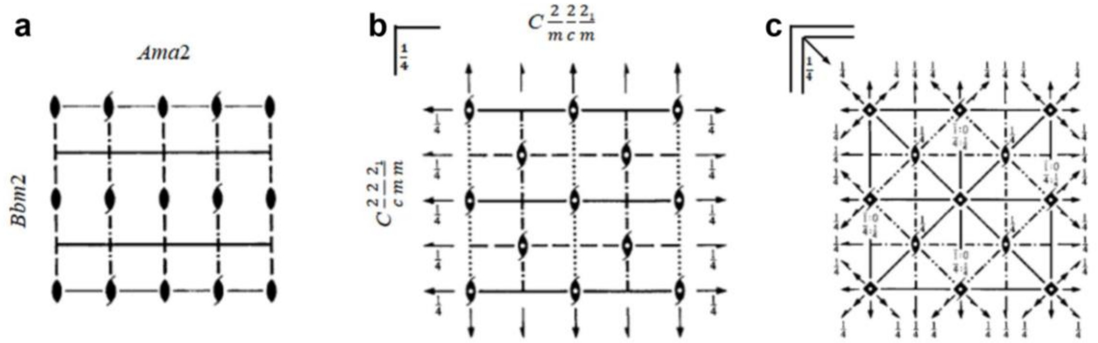

Supplementary Fig. 6 Spatial symmetry operations change from **a** a ferroelectric phase (*Ama*2) to **b** an antiferroelectric phase (*Cmcm*) and **c** a paraelectric phase (*I*4/*mmm*) in **1**.

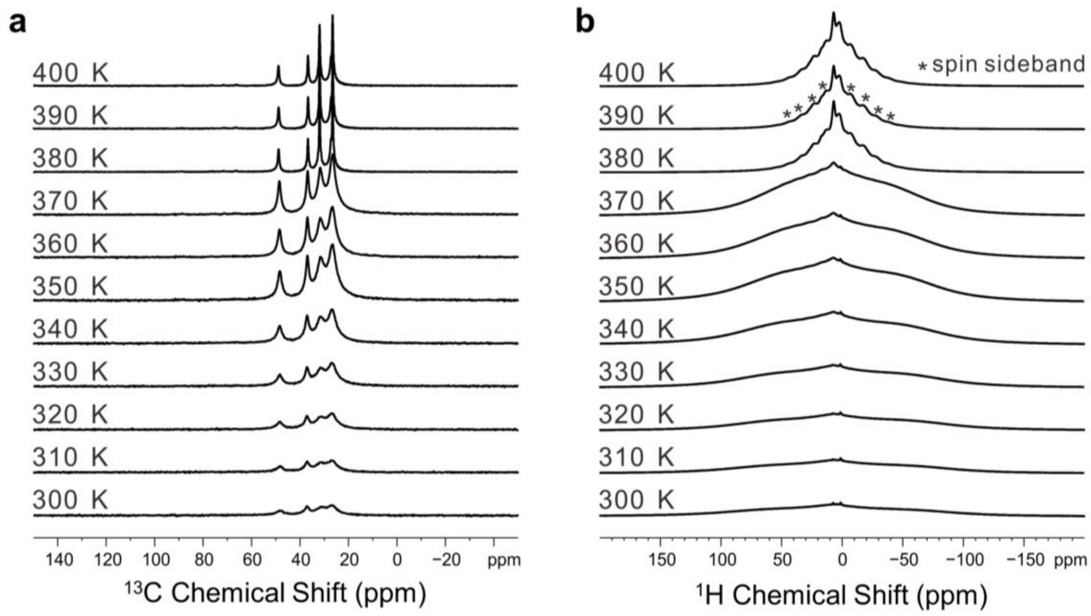

Supplementary Fig. 7 Variable-temperature solid-state nuclear magnetic resonance (NMR) spectra of **1**. **a** ^{13}C NMR and **b** ^{1}H NMR spectra.

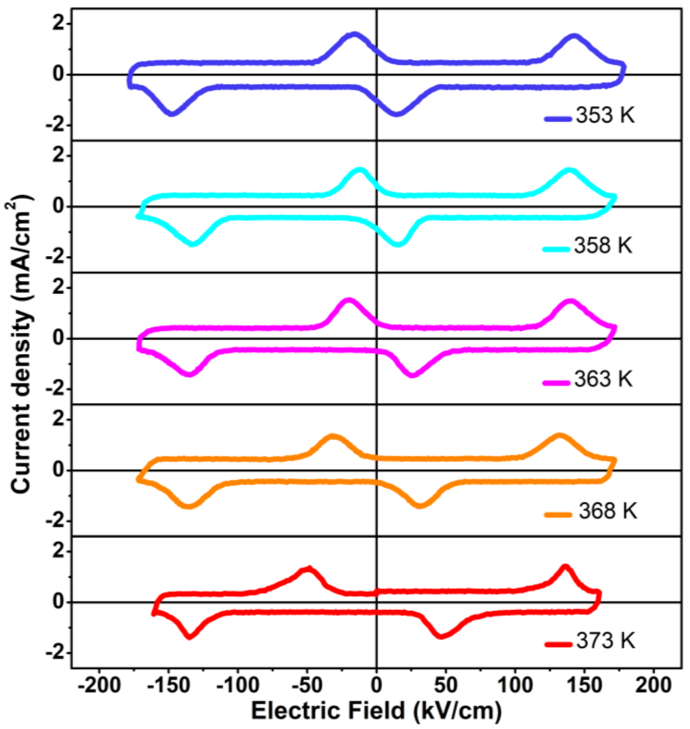

Supplementary Fig. 8 *J*-*E* curves collected at different temperatures.

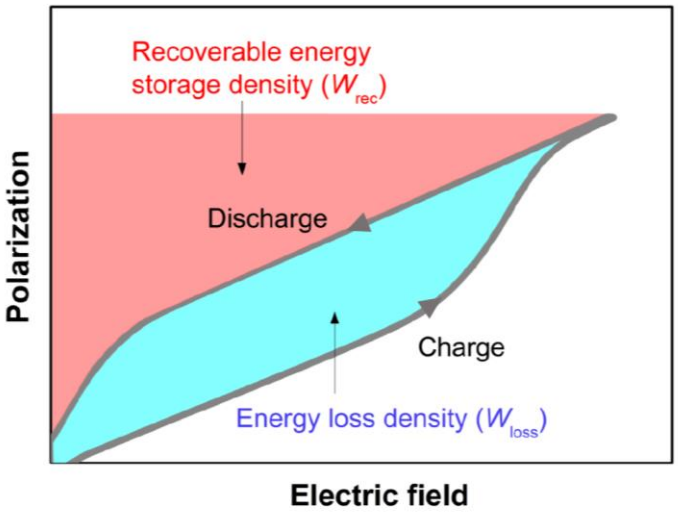

Supplementary Fig. 9 Schematic diagram of the energy-storage mechanisms for AFE materials in a unipolar *P*-*E* loop.

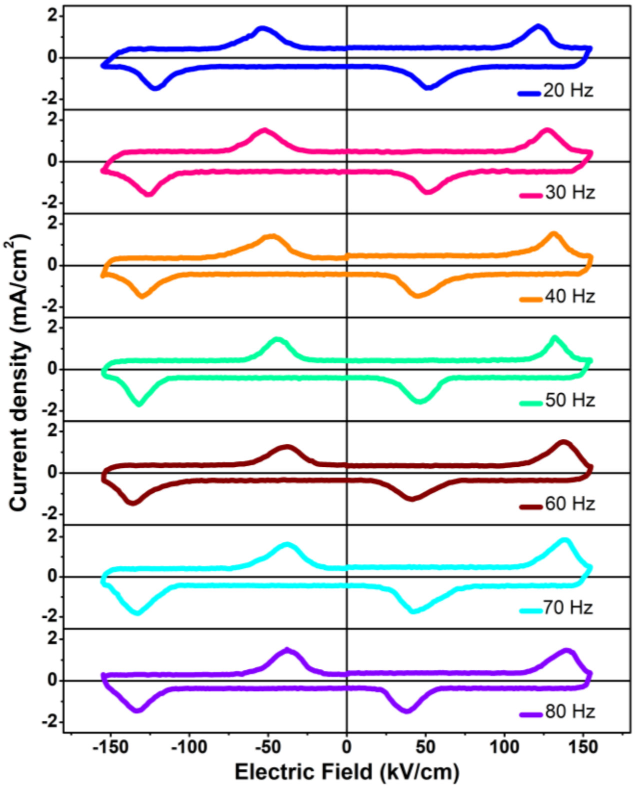

Supplementary Fig. 10 *J*-*E* curves collected at different frequencies.

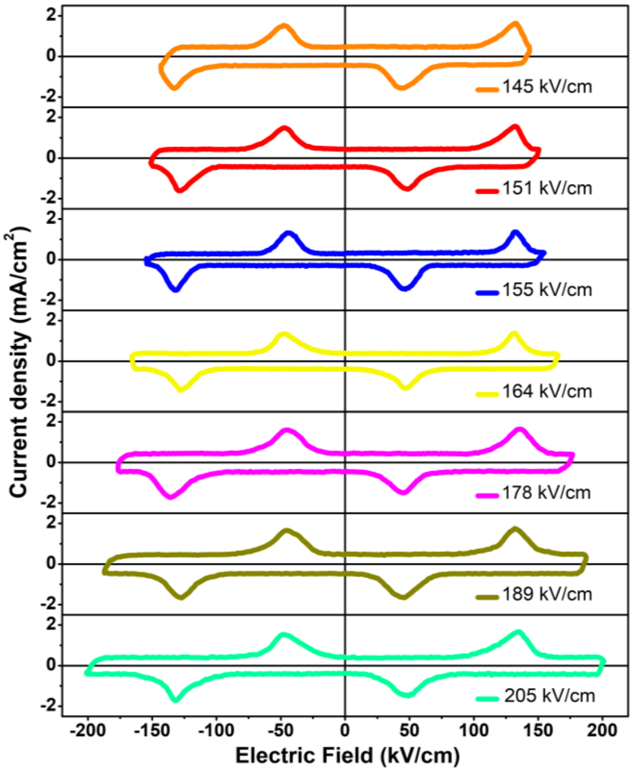

Supplementary Fig. 11 *J*-*E* curves collected at different electric fields.

Supplementary Table 1. Crystal data for **1** collected at low-temperature phase (LTP, 300 K), intermediate-temperature phase (ITP, 360 K) and high-temperature phase (HTP, 390 K), respectively.

	**LTP**	**ITP**	**HTP**
Empirical formula	$C_{14}H_{32}AgBiBr_7CsN_2$	$C_{14}H_{32}AgBiBr_7CsN_2$	$C_{14}H_{32}AgBiBr_7CsN_2$
Formula weight	1237.54	1237.54	1237.54
Temperature/K	300	360	390
Crystal system	Orthorhombic	Orthorhombic	Tetragonal
Space group	*Ama*2	*Cmcm*	*I*4/*mmm*
a (Å)	44.395(3)	8.1597(9)	5.7532(2)
b (Å)	7.9933(5)	8.0368(7)	5.7532(2)
c (Å)	8.1178(5)	44.716(5)	45.062(4)
V (Å^3)	2880.7(3)	2932.4(5)	1491.5(17)
D_{calca}/Mg·m^{-3}	2.853	2.781	2.756
Z	4	4	2
μ (mm^{-1})	17.740	17.144	17.132
F(000)	2240.0	2240.0	1120.0
2Θ range /°	4.59 to 49.97	7.12 to 50.0	7.14 to 54.93
Index ranges	$-50 \le h \le 52$, $-9 \le k \le 9$, $-9 \le l \le 9$	$-9 \le h \le 9$, $-9 \le k \le 9$, $-53 \le l \le 52$	$-6 \le h \le 7$, $-7 \le k \le 5$, $-57 \le l \le 58$
Reflections collected	16012	13102	5601
Independent reflections	2572 [R_{int} = 0.0542, R_{sigma} = 0.0366]	1411 [R_{int} = 0.0703, R_{sigma} = 0.0316]	593 [R_{int} = 0.0414, R_{sigma} = 0.0235]
Data/restraints/parameters	2572/26/123	1411/82/91	593/121/88
Goodness-of-fit on F^2	1.083	1.102	1.117
Final R indexes [$I \ge 2\sigma$ (I)]	R_1 = 0.0741, wR_2 = 0.2106	R_1 = 0.0654, wR_2 = 0.1764	R_1 = 0.0457, wR_2 = 0.0987
Final R indexes [all data]	R_1 = 0.0890, wR_2 = 0.2249	R_1 = 0.0891, wR_2 = 0.1938	R_1 = 0.0598, wR_2 = 0.1063

Supplementary Table 2. Selected Ag/Bi-Br bond lengths of crystal **1** at 300 K.

Atom	Atom	Length/Å	Atom	Atom	Length/Å
Ag/Bi1	Br1	2.805(13)	Ag/Bi1	Br3	2.847(10)
Ag/Bi1^{4}	Br1	2.805(13)	Ag/Bi1^{1}	Br4	2.887(10)
Ag/Bi1	Br2	2.855(10)	Ag/Bi1^{2}	Br4	2.814(10)
Ag/Bi1^{1}	Br2	2.847(10)			

Symmetry transformations used to generate equivalent atoms: ^{1}X, Y-1/2, Z-1/2; ^{2}X, Y, Z-1; ^{4}-X+3/2, Y, Z

Supplementary Table 3. Selected Br-Ag/Bi-Br and Ag/Bi-Br-Ag/Bi bond angles of crystal **1** at 300 K.

Bond	Angle/°	Bond	Angle/°
Br1-Ag/Bi1-Br4^{5}	87.3(4)	Br3-Ag/Bi1-Br2	94.7(4)
Br1-Ag/Bi1-Br4^{9}	88.8(4)	Br3-Ag/Bi1-Br2^{5}	90.3(4)
Br1-Ag/Bi1-Br2	87.4(4)	Br4^{9}-Ag/Bi1-Br4^{5}	175.5(3)
Br1-Ag/Bi1-Br2^{5}	87.7(4)	Br4^{9}-Ag/Bi1-Br2	90.9(14)
Br1-Ag/Bi1-Br3	175.8(3)	Br4^{9}-Ag/Bi1-Br2^{5}	86.9(6)
Br2^{5}-Ag/Bi1-Br4^{5}	90.8(14)	Br4^{9}-Ag/Bi1-Br3	94.9(4)
Br2-Ag/Bi1-Br4^{5}	91.1(6)	Ag/Bi1-Br1-Ag/Bi1^{6}	178.4(9)
Br2^{5}-Ag/Bi1-Br2	174.7(4)	Ag/Bi1^{1}-Br4-Ag/Bi1^{2}	175.5(3)
Br3-Ag/Bi1-Br4^{5}	89.0(4)	Ag/Bi1^{2}-Br2-Ag/Bi1	174.7(4)

Symmetry transformations used to generate equivalent atoms: ^{1}X, Y-3, Z-1; ^{2}X, Y-1/2, Z-1/2; ^{5}X, Y+1/2, Z; ^{6}-X+3/2, Y, Z; ^{9}X, Y, Z+1

Supplementary Table 4. Hydrogen bonds of crystal **1** at 300 K.

D-H···A	d(D-H)	d(H···A)	< DHA	d(D..A)
N1-H1C···Br4^{1}	0.890	2.886	100.50	3.196
N1-H1D···Br3^{1}	0.890	2.898	158.40	3.740
N1-H1E···Br3^{2}	0.890	2.634	165.53	3.503

Symmetry transformations used to generate equivalent atoms: ^{1}X, Y-1/2, Z+1/2; ^{2}X, Y-1, Z

Supplementary Table 5. The applied electric field (E_c) and maximum field amplitude applied (E_m) for various reported molecular antiferroelectrics.

Molecular antiferroelectrics	E_c (kV/cm)	E_m (kV/cm)	Temperature (K)	Ref.
SQA	124	151	295	[1]
[H-55dmbp][Hca]	148	173	295	[1]
TFMBI	12.9	22	295	[1]
DFMBI	67.2	86	295	[1]
TCMBI	49.4	81	295	[1]
γ-FDC	62	127	295	[2]
CPPLA	55	160	295	[2]
PhMDA	110	200	295	[2]
(i-BA)$_2$CsPb$_2$Br$_7$	75	94	298	[3]
(BA)$_2$(EA)$_2$Pb$_3$I$_{10}$	32	60	323	[4]
(isopentylammonium)$_2$CsPb$_2$Br$_7$	8	13	330	[5]
(3-pyrrolinium)CdBr$_3$	25	32	245	[6]
Cu(HCOO)$_2$·4H$_2$O	14	19	233	[7]
La$_2$Cu$_3$[NH(CH$_2$COO)$_2$]$_6$	25	30	350	[8]
CsH$_3$(SeO$_3$)$_2$	45	57	140	[9]
cyclohexylmethylammonium bromide	98	110	310	[10]
**Compound 1**	135	205	353	This work

Supplementary References

1. Horiuchi, S. et al. Strong polarization switching with low-energy loss in hydrogen-bonded organic antiferroelectrics. *Chem. Sci.* **9**, 425-432 (2018).
2. Horiuchi, S. & Ishibashi, S. Large polarization and record-high performance of energy storage induced by a phase change in organic molecular crystals. *Chem. Sci.* **12**, 14198-14206 (2021).
3. Wu, Z. et al. Discovery of an above-room-temperature antiferroelectric in two-dimensional hybrid perovskite. *J. Am. Chem. Soc.*, **141**, 3812-3816 (2019).

4. Han, S. et al. High-temperature antiferroelectric of lead iodide hybrid perovskites. *J. Am. Chem. Soc.*, **141**, 12470-12474 (2019).
5. Li, M. et al. Soft perovskite-type antiferroelectric with giant electrocaloric strength near room temperature. *J. Am. Chem. Soc.* **142**, 20744-20751 (2020).
6. Li, P.-F. et al. Unprecedented ferroelectric-antiferroelectric-paraelectric phase transitions discovered in an organic-inorganic hybrid perovskite. *J. Am. Chem. Soc.* **139**, 8752-8757 (2017).
7. Okada, K. Antiferroelectric phase transition in copper-formate tetrahydrate. *Phys. Rev. Lett.* **15**, 252 (1965).
8. Cui, H. et al. A porous coordination-polymer crystal containing one-dimensional water chains exhibits guest-induced lattice distortion and a dielectric anomaly*Angew. Chem. Int. Ed.* **47**, 3376-3380 (2008).
9. Makita, Y. Phase transition in $CsH_3(SeO_3)_2$. *J. Phys. Soc. Jpn.*, **20**, 1567-1575 (1965).
10. Xu, H. et al. A metal-free molecular antiferroelectric material showing high phase transition temperatures and large electrocaloric effects. *J. Am. Chem. Soc.* **143**, 14379-14385 (2021).
